# Releasing the Kraken

**DOI:** 10.3389/fbinf.2021.808003

**Published:** 2021-12-06

**Authors:** Steven L. Salzberg, Derrick E. Wood

**Affiliations:** ^1^ Department of Biomedical Engineering, Johns Hopkins University, Baltimore, MD, United States; ^2^ Center for Computational Biology, Johns Hopkins University, Baltimore, MD, United States; ^3^ Department of Computer Science, Johns Hopkins University, Baltimore, MD, United States; ^4^ Department of Biostatistics, Johns Hopkins University, Baltimore, MD, United States

**Keywords:** metagenomics, sequence alignment, sequencing indexing, phylogenetic classification, k-mer matching, microbiome

## Abstract

Ten years ago, the dramatic rise in the number of microbial genomes led to an inflection point, when the approach of finding short, exact matches in a comprehensive database became just as accurate as older, slower approaches. The new idea led to a method that was hundreds of times times faster than those that came before. Today, exact k-mer matching is a standard technique at the heart of many microbiome analysis tools.

## Introduction

The field of microbiome research began in the 2000s, at a time when sequencing technology was rapidly getting less costly, and it first became feasible to sequence an environmental sample containing an unknown mixture of organisms. The earliest studies (Venter et al., 2004; Gill et al., 2006) used Sanger sequencing, where sequence lengths were ∼600–800 bp and the cost to sequence a bacterial genome was $50,000 or more. With the advent of Solexa (later Illumina) sequencing technology in 2007, read lengths dropped to just 25 bp, but sequencing costs dropped much faster. Read lengths crept up to 100 bp over the next few years, while costs continued to drop.

In one of the very first microbiome studies to use random shotgun sequencing, published in 2004 (Venter et al., 2004), just under two million reads were generated, averaging 818bp in length. The analysis began by assembling the reads into contigs, and then analyzing only those contigs with sufficient depth of coverage. This yielded 2,226 contigs spanning 30.9Mb, which the authors estimated to represent 1800 different species. The primary tool for identifying species was BLAST (Altschul et al., 1997), which they used to align all bacterial proteins in the NCBI database at the time (∼627 thousand proteins) against the 6-frame translations of all contigs. This was relatively slow, but with just 2,226 contigs, it was feasible.

BLAST remains a powerful tool for determining the best match of any sequence to all known genomes. However, it is far too slow for analysis of modern shotgun sequencing (or even 16S sequencing) experiments. Microbiome experiments can easily generate tens of millions of reads, and it is not unusual to generate well over 100M reads in a single experiment. Any computational step that processes all these reads needs to be very fast.

How fast exactly? Well, in order to process 100M reads in 24 h, a program would have to process over 1,150 reads per second. That is far, far faster than BLAST.

## More Genomes = a new Type of Algorithm

By 2009, there were over 500 complete bacterial genomes, with thousands more in progress (Brady and Salzberg, 2009). As the number of genomes grew, new computational methods were developed to assist with their analysis, and in particular with the core task of assigning a taxonomic label to each read. The label might be the name of a species, genus, family, order, class, or even phylum, depending on how much information was in the sequence. These early methods included: CARMA (Krause et al., 2008), which matched reads to known protein domains, a strategy that worked well when those domains were present, but that had very low sensitivity, only 6% in early experiments; Phylopythia (McHardy et al., 2007), a method that used support vector machines based on oligonucleotide frequencies, and worked best on sequences of 3000 bp or longer; MEGAN (Huson et al., 2007), which used BLAST plus a phylogenetic algorithm; and PhymmBL (Brady and Salzberg, 2009), a method that used interpolated Markov models (IMMs) trained on known species. PhymmBL could handle reads as short as 100 bp, unlike earlier methods, but running thousands of IMMs on each read made it relatively slow. None of these methods were truly superior to BLAST, but they included new ways to assign a read to a taxonomic category, ranging from species to phylum.

Once the number of sequenced species grew sufficiently large, though, it became likelier that most reads in a metagenomics sample would be similar to at least one of the previously-sequenced genomes. This is especially true for well-studied environments such as the human gut microbiome, which many sequencing projects have targeted. With complex environmental samples, more of the species in a sample might not have been seen before, but with over 360,000 prokaryotic genomes available today (of which 25,000 are complete and the rest are in various stages of assembly, as described at NCBI https://www.ncbi.nlm.nih.gov/genome/browse#!/prokaryotes/), the likelihood is far greater now, as compared to the 2000s, that at least one previously-sequenced species is very close to something in a sample.

This observation led us to the idea, back in 2012, that we could forego sequence alignment (e.g., BLAST) and instead identify reads by looking for exact matches of short sequences. Exact matching is far faster than alignment, because it requires a simple table lookup. In its optimal implementation, exact matching requires constant time, while alignment time is at least proportional to the length *n* of the query sequence (and optimal alignment requires *O(n* ^*2*^) time).

For this approach to succeed, we need first to choose a value *k* for the length of our exact matches. *K* needs to be large enough that we can safely assume, in almost all cases, that a match of length *k* is not simply a random match, but rather that the two matching sequences came from the same species, or at least from very closely related species. Thus we can quickly rule out small values such as *k* = 6, because every one of the 4,096 possible 6-mers is likely to be present in most bacterial genomes. At larger values, e.g., *k* = 20, the vast majority of random *k*-mers will not be present in a given bacterial genome, since there are 4^20^ (just over one trillion) 20-mers, and a typical bacterial genome has just one to five million 20-mers.

Thus if we find a 20-base exact match between a read and a genome, there’s a very good chance that the read comes from the same or a similar species. Why not increase the value of *k* even more, which will make this inference more precise (i.e., avoid false positives)? Clearly, for metagenomic analysis the value of *k* cannot be longer than a read. When Kraken first appeared it was not unusual to generate 75 bp reads, so 75 is an initial upper bound for *k*.

There are at least two reasons for reducing the value of the upper bound, though. The first reason is sequencing error: even if the species in a sample exactly matches a known genome, some of the reads will have errors. Illumina technology has a very low error rate, less than 0.5%, so it is reasonable to expect that most 75 bp reads will have one or 0 errors. If the single error is precisely in the middle of the read, then the reads must contain a 37-mer with no errors, suggesting that we might set *k* = 37. The second reason is the simple fact that the species in a microbiome will not be identical to previously-sequenced genomes. We cannot know in advance how similar they will be, but longer values of *k* will mean that we will fail to recognize some species. Thus we can probably choose a value of *k* somewhere between 20 and 37, with higher values yielding lower sensitivity but greater precision.

When we developed Kraken, we initially chose k = 31 for technical reasons: first because larger values of *k* reduce the number of queries to our data structure per sequence; and second because 31 is the largest value of *k* for which we could fit a *k*-mer into a 64-bit integer. In subsequent work, *k* = 31 worked well across a very wide range of databases and experiments, and therefore we kept it as the default value, although the user can adjust *k* when building the Kraken database.

## Speed Matters

When using exact matches instead of a full-blown alignment of reads to genomes, we know that we will never exceed the sensitivity of BLAST. Thus the usefulness of Kraken, and the many competitors that have emerged since, is dependent on its speed. Essentially, we need to find out whether or not a *k*-mer has ever been seen before, and identify where it appeared, as fast as possible. We decided early on that even a single *k*-mer match would be enough to label a read, but that we’d look at every *k*-mer in order to maximize sensitivity. Thus for 100 bp reads with *k* = 31, we would do exactly 70 lookups into our database.

Fortuitously, a very fast *k*-mer counter, called Jellyfish (Marçais and Kingsford, 2011) had recently been developed by our colleagues Guillaume Marçais and Carl Kingsford. Jellyfish counts *k*-mers in a set of DNA sequences (reads or genomes, of any length) and stores the *k*-mer counts in a specialized, highly optimized hash array. It can then query this array very rapidly to report, for any *k*-mer, how often it has occurred.

For metagenomic classification, we do not need to know how often a *k*-mer has appeared, but only what species it occurs in. Every species has a unique taxonomic identifier, available from NCBI, and taking advantage of this, we modified Jellyfish’s output so that for each genome in the database, it would simply store that taxonomy ID next to every *k*-mer in the genome. The only question was what to do for *k*-mers that appear in more than one genome. To keep the data structure from growing too enormous, we wanted to store exactly one ID with each *k*-mer. We solved this problem by using the lowest common ancestor (LCA) of all the genomes in which a *k*-mer appeared. At the time it is building the database, if Kraken encounters a *k*-mer that it has seen before, it queries the NCBI taxonomy and finds the identifier of the LCA, which might be at the genus, family, or higher level.

Thus at the conclusion of the database construction step, Kraken has stored a single taxonomic identifier with every distinct *k*-mer across every genome. The database is stored in a file that is then used for metagenomic classification.

To classify a 100 bp read, Kraken simply walks through it, from position 1 to 70, and looks up all the 31-mers in its database. In most cases, all the *k*-mers are from the same genome and it can simply output that genome’s identifier. If the *k*-mers yield multiple IDs, then Kraken computes the subtree of all the species that it found, and outputs a taxonomy label corresponding to the path in the tree with the most *k*-mers. (Our 2014 paper (Wood and Salzberg, 2014) contains more details.)

This strategy, simple as it is, turned out to be very accurate, with precision of >99% (meaning its false positive rate was <1%) and sensitivity of just over 90%. As expected, BLAST was slightly more sensitive, about 1% higher, and had slightly lower precision, less than 1% lower. (These results were on a simulated dataset in the original study; other results varied but the overall findings were consistent.) One benefit of Kraken’s algorithm is that as the database of known genomes grows, Kraken’s sensitivity has increased over time.

Kraken’s big advantage was speed: in the original paper, we showed that it can classify 1.5 million 92 bp reads per minute (rpm) on a single 2.1 GHz CPU, while Megablast (the “fast” version of BLAST) achieved a rate of 7,143 rpm (Wood and Salzberg, 2014). The fast version of Kraken, Kraken-Q, was even faster, running at 3.9 million rpm, making it >500 times faster than Megablast. Other programs were much slower than Megablast. With slightly longer reads (156bp), Kraken clocked in at 892 K rpm, Kraken-Q ran at 2,842 K rpm, while Megablast processed 2,830 rpm. Thus for the longer reads, Kraken was about 315 times faster and Kraken-Q ran over 1,000 times faster than Megablast.

To illustrate the practical consequences of these speed differences, if we classified a relatively small run of 30 million Illumina reads, Kraken would take about 20 min. Megablast, in contrast, would take 70 h. Analyzing the output of a single run of a current-generation Illumina sequencer, which can generate three billion paired-end reads, would take 100 times longer, which would be less than a day and a half for Kraken, but 10 months with Megablast. This illustrates how the dramatic gains in DNA sequencing efficiency have driven the need for far faster computational methods, even when a solution such as BLAST might initially seem adequate.

## Conclusion

Since we first released Kraken, many other methods have been developed for metagenomics analysis, some of them direct competitors and some that solved related but distinct problems. A recent benchmarking analysis (Ye et al., 2019) compared 20 different metagenomics classifiers on a variety of tasks, and Kraken (along with its successors, KrakenUniq (Breitwieser et al., 2018) and Kraken 2 (Wood et al., 2019)) remains one of the fastest and most accurate methods for identifying reads in a microbiome sample. That study concluded that methods using exact matching of long *k*-mers, the idea pioneered in Kraken, were among the best scoring methods, and that most of the *k*-mer based methods performed similarly to one another.

From an algorithmic perspective, classifying metagenomics reads is a straightforward alignment problem that can be solved by aligning each read to every genome known to science. Optimal solutions to this problem have been known for decades (Fickett, 1984), but they require time that is quadratic in the lengths of the sequences, which is far too slow. As a practical matter, very fast methods are required to keep pace with both the volume of sequence data and the number of sequenced genomes, both of which have been growing at an exponential rate for the past 2 decades. The success of Kraken demonstrates that exact matching of a relatively long subsequence delivers the requisite speed, and with a sufficiently large database of genomes, it also delivers similar accuracy as compared to other methods that are far slower.

## References

[B1] Altschul S. F. Madden T. L. Schäffer A. A. Zhang J. Zhang Z. Miller W. (1997). Gapped BLAST and PSI-BLAST: a New Generation of Protein Database Search Programs. Nucleic Acids Res. 25 (17), 3389–3402. 10.1093/nar/25.17.3389 9254694 PMC146917

[B2] Brady A. Salzberg S. L. (2009). Phymm and PhymmBL: Metagenomic Phylogenetic Classification with Interpolated Markov Models. Nat. Methods 6 (9), 673–676. 10.1038/nmeth.1358 19648916 PMC2762791

[B3] Breitwieser F. P. Baker D. N. Salzberg S. L. (2018). KrakenUniq: Confident and Fast Metagenomics Classification Using Unique K-Mer Counts. Genome Biol. 19 (1), 198. 10.1186/s13059-018-1568-0 30445993 PMC6238331

[B4] Fickett J. W. (1984). Fast Optimal Alignment. Nucleic Acids Res. 12 (1 Pt 1), 175–179. 10.1093/nar/12.1part1.175 6694900 PMC320994

[B5] Gill S. R. Pop M. Deboy R. T. Eckburg P. B. Turnbaugh P. J. Samuel B. S. (2006). Metagenomic Analysis of the Human Distal Gut Microbiome. Science 312 (5778), 1355–1359. 10.1126/science.1124234 16741115 PMC3027896

[B6] Huson D. H. Auch A. F. Qi J. Schuster S. C. (2007). MEGAN Analysis of Metagenomic Data. Genome Res. 17 (3), 377–386. 10.1101/gr.5969107 17255551 PMC1800929

[B7] Krause L. Diaz N. N. Goesmann A. Kelley S. Nattkemper T. W. Rohwer F. (2008). Phylogenetic Classification of Short Environmental DNA Fragments. Nucleic Acids Res. 36 (7), 2230–2239. 10.1093/nar/gkn038 18285365 PMC2367736

[B8] Marçais G. Kingsford C. (2011). A Fast, Lock-free Approach for Efficient Parallel Counting of Occurrences of K-Mers. Bioinformatics 27 (6), 764–770. 10.1093/bioinformatics/btr011 21217122 PMC3051319

[B9] McHardy A. C. Martín H. G. Tsirigos A. Hugenholtz P. Rigoutsos I. (2007). Accurate Phylogenetic Classification of Variable-Length DNA Fragments. Nat. Methods 4 (1), 63–72. 10.1038/nmeth976 17179938

[B10] Venter J. C. Remington K. Heidelberg J. F. Halpern A. L. Rusch D. Eisen J. A. (2004). Environmental Genome Shotgun Sequencing of the Sargasso Sea. Science 304 (5667), 66–74. 10.1126/science.1093857 15001713

[B11] Wood D. E. Lu J. Langmead B. (2019). Improved Metagenomic Analysis with Kraken 2. Genome Biol. 20 (1), 257. 10.1186/s13059-019-1891-0 31779668 PMC6883579

[B12] Wood D. E. Salzberg S. L. (2014). Kraken: Ultrafast Metagenomic Sequence Classification Using Exact Alignments. Genome Biol. 15 (3), R46. 10.1186/gb-2014-15-3-r46 24580807 PMC4053813

[B13] Ye S. H. Siddle K. J. Park D. J. Sabeti P. C. (2019). Benchmarking Metagenomics Tools for Taxonomic Classification. Cell 178 (4), 779–794. 10.1016/j.cell.2019.07.010 31398336 PMC6716367

